# Risk of pneumonia among children with cleft palate before and after palatoplasty: a population-based study

**DOI:** 10.1007/s00431-024-05901-z

**Published:** 2024-12-02

**Authors:** Yotam Eyni, Tomer Kerman, Dana Danino, Aviv Goldbart, Eldad Silberstein

**Affiliations:** 1 Faculty of Health Sciences, Department of Plastic Surgery, Soroka University Medical Center, Ben-Gurion University of the Negev, Be’er Sheva, Israel; 2 Faculty of Health Sciences, Joyce & Irving Goldman Medical School, Ben-Gurion University of the Negev, Be’er-Sheva, Israel; 3 Faculty of Health Sciences, Clinical Research CenterSoroka University Medical Center, Ben-Gurion University of the Negev, Be’er Sheva, Israel; 4 Faculty of Health Sciences, Department of Pediatrics, Saban Children Hospital, Soroka University Medical Center, Ben-Gurion University of the Negev, Be’er Sheva, Israel

**Keywords:** Cleft palate, Infectious diseases, Palatoplasty, Pneumonia

## Abstract

Pneumonia remains the primary cause of mortality among children under age 5. Cleft palate (CP) poses various challenges including velopharyngeal disfunction, potentially increasing rates of pneumonia. However, clinical evidence linking pneumonia to defect is lacking. This study aims to assess the prevalence of pneumonia and lower respiratory tract infections (LRTI) among CP patients through a population-based approach. This retrospective cohort study was conducted using medical records from Clalit Health Services in Israel, 2013–2022, encompassing population of 138,261 infants, including 166 with CP. We investigated the prevalence of pneumonia and other infectious diseases before and after palatoplasty, employing Poisson regression models adjusted for multiple demographic and clinical variables. A sibling comparison study was also conducted, analyzing 129 preoperative and 55 postoperative pairs. The preoperative analysis revealed an increased risk of pneumonia (relative risk [RR]: 5.8, 95% confidence interval (CI): 3.93–8.19), lower respiratory tract infections (LRTI) (*RR*: 1.55, 95% *CI*: 1.12–2.06), and urinary tract infections (UTI) (*RR*: 5.27, 95% *CI*: 3.23–8.03) in children with CP compared to controls. Following surgical repair, a statistically significant but reduced risk for pneumonia persisted up to 5 years (*RR*: 2.55, 95% *CI*: 1.59–3.84). Sibling comparison analyses underscored an elevated pneumonia risk compared to CP siblings preoperatively (*RR*: 7.07, 95% *CI*: 2.48–29.8). Postoperatively, the RR decreased and was no longer statistically significant (*RR*: 1.44, 95% *CI*: 0.49–4.75).

*Conclusion*: Patients with CP are at higher risk for pneumonia, and the magnitude of risk may be reduced after palatoplasty.

**Table:** 

**What is Known:** *• Cleft palate (CP) disrupts the separation of the nasal and oral cavities, leading to food regurgitation into the nasal cavity and theoretically may lead to respiratory tract infections.* *• The association between CP and respiratory tract infections has yet to be assessed thoroughly.*	
**What is New:** *• This population-based study has shown that CP is an independent risk factor for pneumonia.* *• The risk for pneumonia among CP patients is lowered after surgical repair of the defect.*

**Supplementary information:**

The online version contains supplementary material available at 10.1007/s00431-024-05901-z.

## Introduction

Pneumonia remains the leading cause for mortality and morbidity among children below the age of 5 years. Although child mortality rates have declined, pneumonia continues to be the primary cause of death in children beyond the neonatal stage [1–3]. Known risk factors for pneumonia in children are low birth weight, lack of exclusive breastfeeding, crowded living conditions, exposure to indoor air pollution, incomplete immunization, undernutrition, and human immunodeficiency virus infection [4, 5].

Cleft palate (CP) is a common birth defect, affecting approximately 1 in 1000 births worldwide [6]. In southern Israel, CP is more common in the Arab (Bedouin) population than in the Jewish population [7]. CP disrupts the separation of the nasal and oral cavities, leading to issues with swallowing and speech due to velopharyngeal insufficiency [8–11]. Food regurgitation into the nasal cavity is a main factor in these issues, which may increase the risk of pneumonia and other respiratory infections due to micro-aspiration [8, 12]. Difficulty breastfeeding may also raise infection risk, as breastfeeding generally protects against infections, including pneumonia [4, 13, 14].

One study showed that children with CP face higher hospitalization rates for lower respiratory tract infections (LRTI), but it did not specify types of LRTI or focus on pneumonia as the main outcome. Additionally, it only assessed hospitalized cases, overlooking incidents treated as outpatients [15]. Another study in Japan found that cleft lip (CL), independent of CP, is a risk factor for pneumonia. However, due to a small CP sample size, the study could not rule out CP as a risk factor for pneumonia [16]. Despite the physiological reasoning, no study has confirmed the association between CP and higher pneumonia rates, nor the rates of other infectious diseases in CP patients. Furthermore, no research has compared rates of LRTI and other infectious diseases before and after CP repair.

Therefore, in this study, we aimed to assess the prevalence of medical diagnosis of various infectious diseases and particularly pneumonia in CP patients, before and after cleft repair, in both hospital and community out-patient clinics in a population-based study.

## Methods

### Data collection

This retrospective cohort study was conducted using the Clalit Health Services (CHS) data sharing platform, powered by MDClone (https://www.mdclone.com). This platform utilizes advanced algorithms to de-identify and extract data from electronic medical records, ensuring both data quality and patient privacy.

### Study population

The study analyzed birth records from the Southern District, registered with CHS, over the period from January 1, 2013, to December 31, 2022. CHS, the largest health maintenance organization in Israel, provides medical services to approximately 4.8 million individuals, representing 51% of the Israeli population [17]. The Southern District is notable for its ethnic diversity, encompassing both Arab and Jewish populations, and includes a variety of socioeconomic backgrounds and health characteristics. The area spans both urban and rural settings, reflecting the diverse demographic and environmental attributes of southern Israel. The inclusion criteria for the study were birth records from CHS for infants born in the Southern District during the specified study period, resulting in an initial assessment of 143,229 birth records. Exclusion criteria included infants with less than 6 months of documented follow-up within CHS during their first year of life, which could be due to death or termination of membership at CHS. A total of 4968 records were excluded based on these criteria: 4688 were excluded due to end of membership at CHS, and 274 were excluded due to infant death. Data regarding the reason for the exclusion is detailed in Table S28. The final study cohort consisted of 138,261 infants.

### Variable definitions

Cleft palate (CP) was defined as the independent variable in this study. The primary dependent variables were the frequency of infectious respiratory disease events before and after palatoplasty. These diseases included pneumonia, LRTI, upper respiratory tract infections (URTI), and acute otitis media (AOM).

The secondary dependent variables encompassed the frequency of infectious diseases affecting physiological systems outside the respiratory tract, specifically gastroenteritis and urinary tract infections (UTI). These variables served as controls to ascertain if changes in the frequency of respiratory diseases before and after surgery correspond with changes in other types of infections. This helps determine whether the observations are specific to respiratory infections or indicative of a general change in infection susceptibility.

To ensure accurate reporting of distinct infectious disease events, a minimum interval of 2 weeks between diagnoses was mandated. All disease conditions were defined based on their diagnoses in CHS database, using International Classification of Diseases 9 (ICD-9) codes. CP was categorized either by its ICD-9 code or the relevant surgical code. Diagnoses were provided by physicians in hospitals or community clinics, according to established clinical criteria. All specific codes are listed in Table 1.

**Table 1:** Baseline characteristics of study groups before surgery

Characteristic	CP *N* = 166	Control *N* = 138,095	*p*-value
Male, *n* (%)	88 (53)	71,094 (51)	0.7
Ethnicity, *n* (%)			< 0.001
Jew	93 (56)	95,000 (69)	
Arab	62 (37)	26,467 (19)	
Other	11 (6.6)	16,628 (12)	
Socioeconomic level, *n* (%)			0.001
Low	76 (68)	52,915 (52)	
Medium	27 (24)	41,444 (40)	
High	8 (7)	8366 (8)	
Residence location, *n* (%)			0.7
Rural	65 (42)	58,199 (44)	
Suburban	81 (53)	68,403 (52)	
Urban	7 (5)	4573 (4)	
Parental smoking, *n* (%)	47 (30)	43,225 (32)	0.6
Birthweight [g], median (IQR)	2988 (2689, 3309)	3195 (2880, 3500)	< 0.001
Mother age at birth [years], median (IQR)	28 (24.2, 32.7)	28.8 (24.7, 33.3)	0.4
Season of birth, *n* (%)			0.3
Spring	41 (25)	31,576 (23)	
Summer	43 (26)	35,177 (25)	
Autumn	33 (20)	36,163 (26)	
Winter	49 (30)	35,179 (25)	
Birth week, *n* (%)			0.009
< 34	6 (4)	2720 (2)	
34–36	19 (11)	8222 (6)	
≥ 37	141 (85)	127,153 (92)	
Preoperative weight percentile [%], median (IQR)	21 (3, 53)	47 (22, 71)	< 0.001

Comprehensive demographic and clinical data were extracted for each participant including sex, ethnicity, socioeconomic status, and area of residence. The patient’s socioeconomic status was determined by the Israeli Central Bureau of Statistics based on a municipality-level score (1–255), considering 14 factors. Education is as follows: average years of education (ages 25–54) and percentage of individuals (ages 27–54) with an academic degree. Employment and pensions are as follows: percentage of employed individuals (ages 25–54), percentage of women (ages 25–54) without employment income, percentage earning more than twice the average wage, percentage earning below the minimum wage, and percentage of those (aged 20 +) receiving income support or pension supplements. Standard of living is as follows: Average income per capita, vehicle ownership per 100 residents (aged 17 +), average vehicle registration fee (estimated vehicle value), and average number of days spent abroad. Municipalities were grouped into socioeconomic index (SEI) clusters (1–10) and categorized into low (1–3), medium (4–6), or high (7–10) socioeconomic levels [18]. Residence was categorized as urban, suburban, or rural. Additional data collected encompassed parental smoking status, birth weight, maternal age at birth, season of birth, week of birth, preoperative weight percentile, and postoperative weight percentile. Weight percentiles represent a child’s weight relative to a standardized population of the same age and gender, as determined by growth charts. The preoperative weight percentile was defined as the closest weight percentile to 12 months within the age range of 6 to 18 months and the postoperative weight percentile as the closest weight percentile to 4 years within the age range of 30 months to 5 years. Follow-up periods used in this study are detailed in the “Timeframe definitions” section. Detailed information on missing data for covariates is summarized in Table S2.

### Timeframe definitions

The preoperative follow-up period was defined from birth to 1 year of age, based on general clinical practices where most children typically undergo CP repair surgery after one year of age [19].

The postoperative follow-up period extended from 30 months to 5 years of age. This timeframe begins at 30 months, as by this age 90% of the children with CP in this study population had undergone CP repair surgery. This start time was selected to ensure that the follow-up period captured data following the surgery, providing a comprehensive assessment of the patients’ health status postoperatively.

### Preoperative analysis

Initial descriptive analyses were conducted to characterize the study population. This included an assessment of baseline demographic and clinical characteristics. Continuous variables were presented as medians and interquartile ranges (IQR), while categorical variables were described in terms of frequencies and percentages. Continuous variables were compared using *t*-test for normally distributed data and Mann–Whitney test in cases where the distribution departs from normal. Categorical variables were compared using chi-square test, with the use of the Fisher exact test when necessary.

In assessing the risk of infectious diseases preoperatively, a Poisson regression model was applied to estimate the relative risk (RR) for infants with CP compared to those without CP. The models were adjusted consistently for sex, ethnicity, socioeconomic status, birth weight, birth week, and preoperative weight percentile.

All statistical tests were two-tailed, and a *p*-value of less than 0.05 was considered statistically significant. Data were analyzed using R v.3.6.1 software.

### Postoperative analysis

For the postoperative analysis, the study population was selected from the initial cohort of 138,261 infants. Only births with complete follow-up during the postoperative period, defined as 30 months to at least 5 years of age, were included. This required that the infants were born between 2014 and 2018, reached at least the age of 5 years by December 31, 2023, and had not left CHS or died during this period. After applying these criteria, the final population for the postoperative analysis consisted of 68,081 children.

A Poisson regression was employed postoperatively to analyze the RR, with adjustments for sex, ethnicity, socioeconomic status, and postoperative weight percentile.

### Sibling analysis

In addition to the primary analyses, we conducted a sibling comparison study to evaluate the health outcomes of children with CP relative to their non-CP siblings. This analysis utilized a unique matching strategy based on the mother’s ID, ensuring that each CP case was paired with a sibling from the same maternal lineage. Both the preoperative and postoperative sibling populations were selected from the respective preoperative and postoperative cohorts that were previously defined in the study, ensuring consistency in the overall research framework. A significant advantage of this sibling comparison approach is its ability to control shared environmental and genetic factors, which are often confounding variables in observational research. For the preoperative analysis, we identified 129 sibling pairs, and in the postoperative analysis, 55 sibling pairs were included.

To account for the matched nature of the sample, mixed models were employed for comparing the baseline characteristics of the populations. Subsequently, mixed-effects Poisson regression models were applied to assess the RR of infectious diseases pre- and postoperatively, adjusting these models for sex and weight percentile.

### Ethics approval

This retrospective cohort study was approved by the CHS Research Ethics Committee of the Soroka Medical Center Institutional Review Board (approval number: 0339–23-SOR). The study was performed in accordance with the principles of the Declaration of Helsinki.

## Results

This study encompassed a total of 138,261 participants, including 166 individuals identified with CP. Comprehensive demographic and clinical profiles, categorized by research groups, are detailed in Table 1. The distribution of birth weight (median (IQR): CP group 2988 (2689, 3309) g, control group 3195 (2880, 3500), *p* < 0.001) and weight percentile (median (IQR): CP group 21 (3, 53), control group 47 (22, 71), *p* < 0.001) statistically significantly differed between the CP group and controls, with the CP group showing lower values in both measures.

Table 2 presents the incidence per 1000/year and the RR assessments for various infections among infants with CP as compared to the control group preoperatively. In the preoperative analysis, the cohort in this study exhibited an incidence of 50 cases of pneumonia per 1000 children per year, compared to an incidence of 277 cases per 1000 children per year in the cleft palate (CP) group. Using Poisson regression models adjusted for sex, ethnicity, socioeconomic status, birth weight, birth week, and preoperative weight percentile, the analysis revealed a statistically significantly higher incidence of pneumonia (*RR*: 5.8; 95% *CI*: 3.93–8.19), LRTI (*RR*: 1.55; 95% *CI*: 1.12–2.06), and UTI (*RR*: 5.27; 95% *CI*: 3.23–8.03) in the CP group.

**Table 2:** Poisson regression analysis of disease occurrences preoperatively: cleft palate vs. control groups

Disease	CP *N* = 166	Control *N* = 138,095	Adjusted RR for CP (95% CI, *p*-value)
Pneumonia			
Incidence per 1000/year (95% CI)	277 (199–361)	50 (49–51)	5.8 (3.93–8.19, < 0.001)
Median age at first event (months, IQR)	6.96 (2.76, 8.28)	7.68 (5.28, 9.84)	
Median age at event (months, IQR)	7.56 (5.52, 9)	7.8 (5.52, 9.84)	
Lower respiratory tract infection			
Incidence per 1000/year (95% CI)	452 (355–554)	290 (287–293)	1.55 (1.12–2.06, 0.005)
Median age at first event (months, IQR)	4.44 (3.12, 7.32)	5.28 (3.24, 7.8)	
Median age at event (months, IQR)	5.28 (4.08, 7.56)	6 (3.84, 8.16)	
Upper respiratory tract infection			
Incidence per 1000/year (95% CI)	867 (729–1012)	760 (755–765)	1.18 (0.95–1.44, 0.11)
Median age at first event (months, IQR)	5.28 (3, 9)	5.52 (3.12, 8)	
Median age at event (months, IQR)	7.2 (5.04, 9.36)	6.96 (5.04, 8.76)	
Acute otitis media			
Incidence per 1000/year (95% CI)	18 (0–42)	18 (17–19)	1.25 (0.21–3.87, 0.8)
Median age at first event (months, IQR)	8.52 (7.44, 8.76)	8.4 (6.36, 10.2)	
Median age at event (months, IQR)	8.52 (7.44, 8.76)	8.64 (6.6, 10.32)	
Gastroenteritis			
Incidence per 1000/year (95% CI)	133 (78–193)	150 (148–152)	0.67 (0.32–1.22, 0.2)
Median age at first event (months, IQR)	8.28 (4.68, 9.24)	7.8 (5.64, 9.84)	
Median age at event (months, IQR)	8.28 (4.68, 9.24)	8.04 (6, 9.84)	
Urinary tract infection			
Incidence per 1000/year (95% CI)	175 (114–241)	40 (39–41)	5.27 (3.23–8.03, < 0.001)
Median age at first event (months, IQR)	2.16 (0.72, 3.12)	4.32 (1.8, 7.8)	
Median age at event (months, IQR)	3.36 (1.68, 6)	4.8 (2.16, 8.16)	

In the extended follow-up of this cohort, which included 68,081 children, we tracked health outcomes postoperatively until the age of 5 years following CP repair surgery. Table 3 outlines the occurrences of various diseases within this age range, with adjustments accounted for sex, ethnicity, socioeconomic status, and postoperative weight percentile. In the postoperative analysis, the cohort in this study exhibited an incidence of 40 cases of pneumonia per 1000 children per year, compared to an incidence of 128 cases per 1000 children per year in the cleft palate (CP) group. The analysis indicated a persistent, though reduced, increased risk of pneumonia in the CP group postoperatively (*RR*: 2.55; 95% *CI*: 1.59–3.84). For other tested diseases, except for LRTI (*RR*: 1.94, 95% *CI*: 1.14–3.06) and AOM (*RR*: 9.47; 95% *CI*: 5.32–15.4), which showed a statistically significantly higher risk in the CP group, no statistically significant differences were found.

**Table 3:** Poisson regression analysis of disease occurrences postoperatively: cleft palate vs. control groups

Disease	CP *N* = 86	Control *N* = 67,995	Adjusted RR for CP (95% CI, *p*-value)
Pneumonia			
Incidence per 1000/year (95% CI)	128 (58–209)	40 (39–42)	2.55 (1.59–3.84, < 0.001)
Median age at first event (months, IQR)	40.08 (33.6, 50.4)	40.8 (34.8, 48.24)	
Median age at event (months, IQR)	45.48 (38.28, 50.4)	42.24 (36.12, 48.96)	
Lower respiratory tract infection			
Incidence per 1000/year (95% CI)	104 (47–174)	50 (48–51)	1.94 (1.14–3.06, 0.008)
Median age at first event (months, IQR)	44.4 (37.44, 54.12)	39.71 (34.2, 47.76)	
Median age at event (months, IQR)	44.4 (38.76, 54.12)	41.88 (36.12, 48.72)	
Upper respiratory tract infection			
Incidence per 1000/year (95% CI)	337 (221–465)	320 (316–324)	0.77 (0.56–1.02, > 0.087)
Median age at first event (months, IQR)	36.48 (32.16, 45.36)	38.04 (33, 46.08)	
Median age at event (months, IQR)	41.88 (35.16, 47.76)	42.6 (37.32, 48.36)	
Acute otitis media			
Incidence per 1000/year (95% CI)	70 (23–128)	10 (9–11)	9.47 (5.32–15.4, < 0.001)
Median age at first event (months, IQR)	40.56 (37.08, 48.6)	39.48 (34.2, 48.84)	
Median age at event (months, IQR)	44.64 (41.52, 48.6)	41.52 (35.76, 49.32)	
Gastroenteritis			
Incidence per 1000/year (95% CI)	70 (23–128)	50 (48–52)	1.56 (0.88–2.53, 0.1)
Median age at first event (months, IQR)	38.04 (34.32, 41.88)	40.68 (34.8, 48.48)	
Median age at event (months, IQR)	39.6 (35.28, 46.8)	41.88 (35.76, 48.96)	
Urinary tract infection			
Incidence per 1000/year (95% CI)	23 (0–58)	20 (19–21)	1.16 (0.36–2.71, 0.8)
Median age at first event (months, IQR)	41.28 (39.84, 44.52)	42 (35.76, 50.16)	
Median age at event (months, IQR)	41.28 (39.84, 44.52)	43.56 (37.44, 50.88)	

In this investigation, we conducted a detailed sibling comparison analysis, which comprised two distinct cohorts: the preoperative group with 129 sibling pairs and the postoperative group with 55 sibling pairs. The adjusted mixed-effects Poisson regression analysis revealed a statistically significant increase in the risk of pneumonia among siblings with CP prior to surgery (*RR*: 7.07; 95% *CI*: 2.48–29.8). Postoperatively, the RR decreased and was no longer statistically significant (*RR*: 1.44; 95% *CI*: 0.49–4.75). The specific occurrences of diseases both preoperatively and postoperatively are comprehensively documented in Tables 4 and 5, respectively.

**Table 4:** Poisson regression analysis of disease occurrences preoperatively: cleft palate siblings vs. non-cleft palate siblings

Disease	CP *N* = 129	Control *N* = 129	Adjusted RR for CP (95% CI, *p*-value)
Pneumonia			
Incidence per 1000/year (95% CI)	287 (202–380)	31 (8–62)	7.07 (2.48–29.8, 0.001)
Median age at first event (months, IQR)	7.44 (3.12, 9)	7.44 (6.24, 8.28)	
Median age at event (months, IQR)	7.56 (5.52, 9.36)	7.44 (6.24, 8.28)	
Lower respiratory tract infection			
Incidence per 1000/year (95% CI)	457 (341–581)	302 (209–403)	1.3 (0.78–2.19, 0.3)
Median age at first event (months, IQR)	4.32 (3.12, 7.56)	4.92 (3, 6.24)	
Median age at event (months, IQR)	4.92 (4.08, 7.92)	5.16 (3.36, 6.96)	
Upper respiratory tract infection			
Incidence per 1000/year (95% CI)	868 (713–1031)	713 (574–860)	1.1 (0.78–1.57, 0.11)
Median age at first event (months, IQR)	5.64 (3, 9.36)	4.8 (2.76, 8.16)	
Median age at event (months, IQR)	7.32 (5.04, 9.72)	6.48 (4.44, 8.76)	
Acute otitis media			
Incidence per 1000/year (95% CI)	23 (0–54)	23 (0.54)	1.1 (0.19–6.35, > 0.9)
Median age at first event (months, IQR)	8.52 (7.44, 8.76)	6 (5.76, 6.24)	
Median age at event (months, IQR)	8.52 (7.44, 8.76)	6.24 (5.88, 6.6)	
Gastroenteritis			
Incidence per 1000/year (95% CI)	147 (85–217)	240 (163–326)	0.63 (0.31–1.29, 0.2)
Median age at first event (months, IQR)	8.4 (6.6, 9.36)	6.6 (5.76, 7.56)	
Median age at event (months, IQR)	8.4 (6.6, 9.36)	7.2 (5.88, 8)	
Urinary tract infection			
Incidence per 1000/year (95% CI)	132 (70–202)	93 (47–147)	1.25 (0.51–3.27, 0.6)
Median age at first event (months, IQR)	2.88 (1.2, 3.12)	6.72 (2.64, 9.96)	
Median age at event (months, IQR)	3.48 (2.88, 5.28)	6.12 (3, 9.96)

**Table 5:** Poisson regression analysis of disease occurrences postoperatively: cleft palate siblings vs. non-cleft palate siblings

Disease	CP *N* = 55	Control *N* = 55	Adjusted RR for CP (95% *CI*, *p*-value)
Pneumonia			
Incidence per 1000/year (95% CI)	91 (18–182)	40 (0–91)	1.44 (0.49–4.75, 0.5)
Median age at first event (months, IQR)	41.76 (32.16, 46.8)	51.84 (49.32, 55.32)	
Median age at event (months, IQR)	45.36 (37.08, 49.08)	51.96 (49.44, 5.32)	
Lower respiratory tract infection			
Incidence per 1000/year (95% CI)	100 (18–200)	36 (0–91)	1.72 (0.56–6.45, 0.4)
Median age at first event (months, IQR)	48.84 (37.44, 54.72)	48.72 (45.84, 56.4)	
Median age at event (months, IQR)	48.84 (3.34, 4.56)	52 (45.84, 56.4)	
Upper respiratory tract infection			
Incidence per 1000/year (95% CI)	364 (218–527)	255 (127–400)	0.73 (0.42–1.25, 0.2)
Median age at first event (months, IQR)	36.24 (32.16, 46.2)	43.68 (37.8, 47.04)	
Median age at event (months, IQR)	41.16 (35.04, 48.48)	44.52 (41.88, 48.36)	
Acute otitis media			
Incidence per 1000/year (95% CI)	73 (18–145)	–	–
Median age at first event (months, IQR)	38.88 (35.4, 42.12)	–	
Median age at event (months, IQR)	42.12 (39.72, 44.64)	–	
Gastroenteritis			
Incidence per 1000/year (95% CI)	60 (0–127)	30 (0–90)	2.07 (0.56–9.76, 0.3)
Median age at first event (months, IQR)	37.56 (33.36, 40.08)	34.32 (33.96, 39.72)	
Median age at event (months, IQR)	3.34 (3.27, 3.36)	34.32 (33.96, 41.16)	
Urinary tract infection			
Incidence per 1000/year (95% CI)	18 (0–55)	–	–
Median age at first event (months, IQR)	46.8 (44.52, 49.08)	–	
Median age at event (months, IQR)	46.8 (44.52, 49.08)	–	

## Discussion

This study exhibits an incidence of 40–50 cases of pneumonia per 1000 children per year among controls under 5 years old and an incidence of 128–277 cases of pneumonia per 1000 children per year among CP patients under 5 years old. The known incidence of pneumonia worldwide under 5 years old is 30 cases per 1000 children per year in industrialized countries [20]. This population-based study has shown, for the first time, that children with CP are at higher risk for pneumonia compared to controls after adjusting for sex, ethnicity, socioeconomic status, birth weight, birth gestational age, and weight percentile. The highest risk for pneumonia was in the preoperative period.

Palatoplasty has been shown to improve feeding abilities in children with CP, apparently by improving velopharyngeal function [10]. Therefore, we also conducted an analysis to assess whether the risk for pneumonia is reduced after repair of the defect by surgery. Interestingly, the higher risk for pneumonia was persistent but reduced after the age of palatoplasty and up to 5 years old. These findings may suggest that palatoplasty reduces the rate of pneumonia in these patients. Further study that investigates swallowing function in these patients is required to evaluate the weight of palate defect repair on the risk for pneumonia.

To eliminate confounding variables that may increase rates of pneumonia that we could not assess, such as air pollution exposure, crowding at residence, vaccination status, and other unknown shared environmental and genetic factors, we conducted an analysis comparing the CP group of patients to their own sibling without CP. Presumably, the sibling group shares most of these unknown mediators with the CP patients’ group. Similar results have been shown with higher risk for pneumonia in CP patients versus their non-CP siblings in the first year of life and after surgery up to 5 years old. This is the first study to have conducted such an analysis to establish the connection between CP and pneumonia.

To our knowledge, this is the largest study that evaluates infectious diseases rates and pneumonia specifically among patients with CP. Another study, which assessed prevalence of hospitalization of children with different congenital defects, has shown higher rates of hospitalization of children with CP for LRTI [15]. Our findings strengthen these results by including inpatient and outpatient diagnosis of pneumonia and LRTI.

It is reasonable that CP patients are at higher risk for different kinds of common infectious diseases due to lower birthweight and difficulties with breastfeeding [21, 22]. However, pneumonia is the only infectious disease that has been found to be at higher rates in all analyses of this study, at different ages and comparing to different controls. Nevertheless, other infectious diseases were also at higher rates in CP patients versus controls. For example, higher rates of AOM among CP patients were found between 16 months and 5 years old of age in both general and siblings analysis. It is well established that CP is a risk factor for AOM. It is thought that insufficient clearing of the nasal cavity is related to inability of the Eustachian tube to drain adequately liquids from the ear cavity, therefore, causing effusion and stasis which elevates the rates of colonization and infections [12, 23, 24]. In the first year of life, the prevalence of AOM was not higher among CP patients versus controls. Interestingly, it is known that recurrence of AOM and chronic AOM starts to develop later in life in CP infants, and it is common throughout childhood into adolescence [25]. It may be explained by the time it takes for stasis and congestion to cause damage, worsening eustachian tube function and causing more stasis and eventually recurrent effusion and infection [26, 27]. UTI was found to be more prevalent in CP patients during their first year of life only compared to general controls but not compared to the non-CP siblings control group. Recent data show that children with lower respiratory tract infection admitted to pediatric wards suffer frequently from secondary bacterial UTI [28], and this might be the case in many of the children in this cohort. It might also be explained by higher rates of hospitalization and catheterizations in these patients that may increase the risk for UTI [29, 30]. Higher rates of UTI and AOM in CP patients suggest they are vulnerable to other kinds of infectious diseases as well. But the fact that solely pneumonia was at higher risk in both analyses, compared to the non-CP siblings group and to the general controls, suggests that pneumonia is directly related to the anatomical anomaly among these patients.

There are some limitations in this study. The lack of swallowing evaluation, breastfeeding status, and severity of defect in CP patients of this study is a significant limitation. This study strengthens the hypothesis that velopharyngeal disfunction is a main contributor to higher rates of pneumonia in CP patients. But this concept should be evaluated by assessing the association between swallowing function itself and pneumonia rates. Other important risk factors for pneumonia were also missing in this study, such as number of siblings at residence and vaccination status. Since most siblings share these variables, the CP patients versus non-CP siblings analysis were done to mitigate these possible confounding factors. The ideal method to determine a definition of pneumonia as a variable is by adequate clinical, radiological, and laboratory findings. But using only the ICD-9 diagnosis, as in this study, is common and has high specificity for cases of pneumonia [31]. Obviously, it also enabled the large scale of analysis in this study and makes it simple to external validate.

## Conclusion

Patients with CP are at higher risk for pneumonia and other infectious diseases. We hypothesize that velopharyngeal insufficiency in these patients contributes to higher rates of pneumonia, but more research on swallowing function and aspiration rates is essential to validate this hypothesis. We also hypothesize that palatoplasty may contribute to reduction in the risk of pneumonia. By identifying these children as a high-risk group, healthcare providers can prioritize early diagnosis and treatment, potentially reducing the incidence and severity of pneumonia in this population.

## Supplementary information

Below is the link to the electronic supplementary material.

Supplementary file1 (DOCX 60 KB)

## Data Availability

Data are available on request. For requests and more information, please contact the corresponding author.

## Abbreviations

AOM: Acute otitis media
CHS: Clalit Health Services
CI: Confidence interval
CL: Cleft lip
CP: Cleft palate
ICD-9: International Classification of Diseases 9
IQR: Interquartile range
LRTI: Lower respiratory tract infection
RR: Relative risk
URTI: Upper respiratory tract infection
