# Supplementary material for: Risk of pneumonia among children with cleft palate before and after palatoplasty: a population-based study
Source: Eur J Pediatr. 2024 Dec 2;184(1):56. doi: 10.1007/s00431-024-05901-z (PMC11611938; doi:10.1007/s00431-024-05901-z)
Supplement: Supplementary file 1 — Supplementary file1 (DOCX 60 KB) [file 431_2024_5901_MOESM1_ESM.docx]

**Table S1: List of ICD-9 Codes**

| **Description** | **ICD9 code** | **Disease** |
| --- | --- | --- |
| **Independent variable** | | |
| Cleft palate | 749.0 | CP |
| Cleft palate with cleft lip | 749.2 |  |
| Procedure code: Correction of cleft palate | 27.62 |  |
| **Primary dependent variables** | | |
| Pneumococcal pneumonia [Streptococcus pneumoniae pneumonia] | 481.X | Pneumonia |
| Other bacterial pneumonia | 482.X |  |
| Pneumonia due to other specified organism | 483.X |  |
| Pneumonia in infectious diseases classified elsewhere | 484.X |  |
| Pneumonia, organism unspecified | 486.X |  |
| Pneumonitis due to solids and liquids | 507.X |  |
| Empyema | 510.X |  |
| Pleurisy | 511.X |  |
| Abscess of lung and mediastinum | 513.X |  |
| Acute bronchitis and bronchiolitis | 466.X | LRTI |
| Viral pneumonia | 480.X |  |
| Influenza | 487.X |  |
| Influenza due to certain identified influenza viruses | 488.X |  |
| Bronchitis, not specified as acute or chronic | 490.X |  |
| Acute upper respiratory infections of other multiple sites | 465.8 | URTI |
| Acute upper respiratory infections of unspecified site | 465.9 |  |
| Allergic rhinitis | 477.X |  |
| Other diseases of upper respiratory tract | 478.X |  |
| Acute suppurative otitis media | 382.0X | AOM |
| **Secondary dependent variables** | | |
| Ill-defined intestinal infections | 009.X | Gastroenteritis |
| Infections of kidney | 590.X | UTI |
| Cystitis | 595.X |  |
| Urinary tract infection, site not specified | 599.0 |  |
| Urinary tract infection of newborn | 771.82 |  |

**Table S2: Missing values for each variable**

| **Variable** | **Number of missing** | | | | **Types of variable** |
| --- | --- | --- | --- | --- | --- |
|  | **CP** | | **Control** | |  |
|  | N | % | N | % |  |
| Sex | 0 | 0 | 0 | 0 | Categorical |
| Ethnicity | 0 | 0 | 0 | 0 | Categorical |
| Socioeconomic level | 55 | 33.13 | 35,370 | 25.61 | Categorical |
| Residence location | 13 | 7.83 | 6,920 | 5.01 | Categorical |
| Parental smoking | 9 | 5.42 | 3,017 | 2.18 | Categorical |
| Birthweight | 0 | 0 | 3 | 0.002 | Continuous |
| Mother age at birth | 0 | 0 | 0 | 0 | Continuous |
| Season of birth | 0 | 0 | 0 | 0 | Categorical |
| Birth week | 0 | 0 | 0 | 0 | Categorical |
| Preoperative weight percentile | 44 | 26.5 | 47,129 | 34.13 | Continuous |
| Postoperative weight percentile | 31 | 36 | 28,598 | 42 | Continuous |
| CP | 0 | 0 | 0 | 0 | Categorical |
| Pneumonia | 0 | 0 | 0 | 0 | Categorical |
| LRTI | 0 | 0 | 0 | 0 | Categorical |
| URTI | 0 | 0 | 0 | 0 | Categorical |
| AOM | 0 | 0 | 0 | 0 | Categorical |
| Gastroenteritis | 0 | 0 | 0 | 0 | Categorical |
| UTI | 0 | 0 | 0 | 0 | Categorical |

**Table S3: Baseline Characteristics of Study Groups After Surgery**

| **Characteristic** | **CP,**  N = 86 | **Control,**  N = 67,995 | **p-value** |
| --- | --- | --- | --- |
| **Male sex, n (%)** | 52 (60) | 35,007 (51) | 0.1 |
| **Ethnicity, n (%)** |  |  | <0.001 |
| Jew | 50 (58) | 52,600 (77) |  |
| Arab | 36 (42) | 14,601 (21) |  |
| Other | – | 794 (1.2) |  |
| **Socioeconomic level, n (%)** |  |  | 0.14 |
| Low | 32 (57) | 24,143 (47) |  |
| Medium | 17 (30) | 22,539 (43) |  |
| High | 7 (13) | 5,146 (10) |  |
| **Residence location, n (%)** |  |  | 0.2 |
| Rural | 28 (35) | 27,913 (43) |  |
| Suburban | 46 (58) | 33,521 (52) |  |
| Urban | 6 (7.5) | 3,193 (5) |  |
| **Parental smoking status, n (%)** | 24 (30) | 22,543 (34) | 0.5 |
| **Birthweight [gr], median (IQR)** | 3,050 (2,771, 3,390) | 3,190 (2,875, 3,500) | 0.018 |
| **Mother age at birth [years], median (IQR)** | 28.2 (24, 32.7) | 29 (24.9, 33.3) | 0.4 |
| **Season of birth, n (%)** |  |  | 0.068 |
| Spring | 18 (21) | 17,073 (25) |  |
| Summer | 31 (36) | 17,016 (25) |  |
| Autumn | 14 (16) | 16,571 (25) |  |
| Winter | 23 (27) | 17,335 (25) |  |
| **Birth week, n (%)** |  |  | 0.2 |
| < 34 | 4 (4.7) | 1,403 (2.1) |  |
| 34-36 | 5 (5.8) | 4,093 (6) |  |
| ≥ 37 | 77 (90) | 62,499 (92) |  |
| **Postoperative weight percentile [%], median (IQR)** | 27 (14, 52) | 44 (21, 67) | 0.004 |

**Table S4: Baseline Characteristics of Sibling Groups Before Surgery**

| **Characteristic** | **Siblings with**  **CP,**  **N = 129** | **Siblings without**  **CP,**  **N = 129** | **p-value** |
| --- | --- | --- | --- |
| **Year of birth, median (IQR)** | 2018 (2015, 2020) | 2017 (2014, 2020) | 0.1 |
| **Male, n (%)** | 68 (53) | 57 (44) | 0.2 |
| **Ethnicity, n (%)** |  |  | >0.9 |
| Jew | 65 (50) | 65 (50) |  |
| Arab | 54 (42) | 54 (42) |  |
| Other | 10 (8) | 10 (8) |  |
| **Socioeconomic level, n (%)** |  |  | >0.9 |
| Low | 63 (74) | 62 (71) |  |
| Medium | 18 (21) | 21 (24) |  |
| High | 4 (5) | 4 (5) |  |
| **Residence location, n (%)** |  |  | >0.9 |
| Rural | 55 (46) | 56 (46) |  |
| Suburban | 60 (50) | 61 (50) |  |
| Urban | 3 (2.5) | 4 (3) |  |
| **Parental smoking, n (%)** | 40 (31) | 40 (31) | >0.9 |
| **Birthweight [gr], median (IQR)** | 2,965 (2,715, 3,305) | 3,100 (2,870, 3,385) | 0.15 |
| **Mother age at birth [years], median (IQR)** | 27.9 (24.2, 32.5) | 27.7 (23.7, 31.6) | 0.4 |
| **Season of birth, n (%)** |  |  | 0.13 |
| Spring | 33 (26) | 21 (16) |  |
| Summer | 34 (26) | 37 (29) |  |
| Autumn | 26 (20) | 39 (30) |  |
| Winter | 36 (28) | 32 (25) |  |
| **Birth week, n (%)** |  |  | 0.2 |
| < 34 | 4 (3) | 4 (3) |  |
| 34-36 | 16 (12) | 8 (6) |  |
| ≥ 37 | 109 (85) | 117 (91) |  |
| **Preoperative weight percentile [%], median (IQR)** | 21 (3, 52) | 52 (22, 75) | <0.001 |

**Table S5: Baseline Characteristics of Sibling Groups After Surgery**

| **Characteristic** | **Siblings**  **with CP,**  **N = 55** | **Siblings**  **without CP,**  **N = 55** | **p-value** |
| --- | --- | --- | --- |
| **Year of birth, median (IQR)** | 2015 (2013, 2016) | 2015 (2014, 2017) | 0.3 |
| **Male sex, n (%)** | 34 (62) | 24 (44) | 0.055 |
| **Ethnicity, n (%)** |  |  | >0.9 |
| Jew | 30 (55) | 30 (55) |  |
| Arab | 25 (45) | 25 (45) |  |
| **Socioeconomic level, n (%)** |  |  | 0.9 |
| Low | 26 (63) | 25 (68) |  |
| Medium | 11 (27) | 8 (22) |  |
| High | 4 (10) | 4 (10) |  |
| **Residence location, n (%)** |  |  | >0.9 |
| Rural | 20 (41) | 19 (37) |  |
| Suburban | 27 (55) | 30 (59) |  |
| Urban | 2 (4) | 2 (4) |  |
| **Parental smoking status, n (%)** | 17 (31) | 17 (31) | >0.9 |
| **Birthweight [gr], median (IQR)** | 3,050 (2,705, 3,413) | 3,140 (2,725, 3,465) | 0.8 |
| **Mother age at birth [years], median (IQR)** | 27.9 (24, 32.2) | 27.3 (23.3, 32.5) | 0.8 |
| **Season of birth, n (%)** |  |  | 0.7 |
| Spring | 12 (22) | 9 (16) |  |
| Summer | 19 (35) | 16 (29) |  |
| Autumn | 10 (18) | 12 (22) |  |
| Winter | 14 (25) | 18 (33) |  |
| **Birth week, n (%)** |  |  | 0.8 |
| < 34 | 3 (5.5) | 2 (3.6) |  |
| 34-36 | 3 (5.5) | 2 (3.6) |  |
| ≥ 37 | 49 (89) | 21 (9.3) |  |
| **Postoperative weight percentile [%], median (IQR)** | 30 (11, 50) | 43 (23, 60) | 0.13 |

**Table S6: Multivariable Poisson Regression Analysis of Pneumonia Occurrences Preoperatively**

| **Characteristic** | **RR** | **95% CI** | **p-value** |
| --- | --- | --- | --- |
| CP | 5.8 | 3.93-8.19 | <0.001 |
| Male sex | 1.35 | 1.26-1.44 | 0.004 |
| Ethnicity |  |  |  |
| Jew | Reference |  |  |
| Arab | 1.18 | 1.07-1.29 | <0.001 |
| Other | 1.42 | 1.25-1.44 | <0.001 |
| Socioeconomic level |  |  |  |
| Low | Reference |  |  |
| Medium | 1.04 | 0.97-1.13 | 0.3 |
| High | 0.93 | 0.83-1.05 | 0.3 |
| Birthweight | 1 | 1-1 | 0.3 |
| Birth week | 0.95 | 0.93-0.97 | <0.001 |
| Preoperative weight percentile | 1 | 1-1 | 0.004 |

**Table S7: Multivariable Poisson Regression Analysis of Lower Respiratory Tract Infection Occurrences Preoperatively**

| **Characteristic** | **RR** | **95% CI** | **p-value** |
| --- | --- | --- | --- |
| CP | 1.55 | 1.12-2.06 | 0.005 |
| Male sex | 1.33 | 1.3-1.37 | <0.001 |
| Ethnicity |  |  |  |
| Jew | Reference |  |  |
| Arab | 1.35 | 1.3-1.41 | <0.001 |
| Other | 1.04 | 0.98-1.11 | 0.2 |
| Socioeconomic level |  |  |  |
| Low | Reference |  |  |
| Medium | 0.78 | 0.76-0.81 | <0.001 |
| High | 0.6 | 0.57-0.64 | <0.001 |
| Birthweight | 1 | 1-1 | 0.061 |
| Birth week | 0.96 | 0.95-0.97 | <0.001 |
| Preoperative weight percentile | 1 | 1-1 | <0.001 |

**Table S8: Multivariable Poisson Regression Analysis of Upper Respiratory Tract Infection Occurrences Preoperatively**

| **Characteristic** | **RR** | **95% CI** | **p-value** |
| --- | --- | --- | --- |
| CP | 1.18 | 0.95-1.44 | 0.11 |
| Male sex | 1.13 | 1.11-1.15 | <0.001 |
| Ethnicity |  |  |  |
| Jew | Reference |  |  |
| Arab | 1.42 | 1.39-1.45 | <0.001 |
| Other | 0.87 | 0.84-0.91 | <0.001 |
| Socioeconomic level |  |  |  |
| Low | Reference |  |  |
| Medium | 0.93 | 0.91-0.95 | <0.001 |
| High | 0.95 | 0.92-0.98 | 0.001 |
| Birthweight | 1 | 1-1 | <0.001 |
| Birth week | 0.99 | 0.98-0.99 | <0.001 |
| Preoperative weight percentile | 1 | 1-1 | 0.029 |

**Table S9: Multivariable Poisson Regression Analysis of Acute Otitis Media Occurrences Preoperatively**

| **Characteristic** | **RR** | **95% CI** | **p-value** |
| --- | --- | --- | --- |
| CP | 1.25 | 0.21-3.87 | 0.8 |
| Male sex | 1.25 | 1.12 | <0.001 |
| Ethnicity |  |  |  |
| Jew | Reference |  |  |
| Arab | 1.07 | 0.9-1.27 | 0.4 |
| Other | 1.34 | 1.09-1.27 | 0.4 |
| Socioeconomic level |  |  |  |
| Low | Reference |  |  |
| Medium | 1.43 | 1.26-1.63 | <0.001 |
| High | 1.43 | 1.18-1.72 | <0.001 |
| Birthweight | 1 | 1-1 | 0.14 |
| Birth week | 0.98 | 0.94-1.01 | 0.2 |
| Preoperative weight percentile | 1 | 1-1 | 0.4 |

**Table S10: Multivariable Poisson Regression Analysis of Gastroenteritis Occurrences Preoperatively**

| **Characteristic** | **RR** | **95% CI** | **p-value** |
| --- | --- | --- | --- |
| CP | 0.67 | 0.32-1.22 | 0.2 |
| Male sex | 1.16 | 1.11-1.21 | <0.001 |
| Ethnicity |  |  |  |
| Jew | Reference |  |  |
| Arab | 1.29 | 1.22-1.36 | <0.001 |
| Other | 1.13 | 1.03-1.23 | 0.006 |
| Socioeconomic level |  |  |  |
| Low | Reference |  |  |
| Medium | 0.69 | 0.66-0.72 | <0.001 |
| High | 0.66 | 0.61-0.72 | <0.001 |
| Birthweight | 1 | 1-1 | 0.3 |
| Birth week | 1.02 | 1-1.03 | 0.024 |
| Preoperative weight percentile | 1 | 1-1 | 0.007 |

**Table S11: Multivariable Poisson Regression Analysis of Urinary Tract Infection Occurrences Preoperatively**

| **Characteristic** | **RR** | **95% CI** | **p-value** |
| --- | --- | --- | --- |
| CP | 5.27 | 3.23-8.03 | <0.001 |
| Male sex | 0.35 | 0.32-0.38 | <0.001 |
| Ethnicity |  |  |  |
| Jew | Reference |  |  |
| Arab | 1.66 | 1.5-1.84 | <0.001 |
| Other | 1.08 | 0.91-1.26 | 0.4 |
| Socioeconomic level |  |  |  |
| Low | Reference |  |  |
| Medium | 1.13 | 1.03-1.23 | 0.009 |
| High | 0.87 | 0.75-1.01 | 0.07 |
| Birthweight | 1 | 1-1 | 0.8 |
| Birth week | 0.93 | 0.91-0.95 | <0.001 |
| Preoperative weight percentile | 1 | 1-1 | 0.4 |

**Table S12: Multivariable Poisson Regression Analysis of Pneumonia Occurrences Postoperatively**

| **Characteristic** | **RR** | **95% CI** | **p-value** |
| --- | --- | --- | --- |
| CP | 2.55 | 1.59-3.84 | <0.001 |
| Male sex | 0.95 | 0.9-1 | 0.039 |
| Ethnicity |  |  |  |
| Jew | Reference |  |  |
| Arab | 1.14 | 1.06-1.22 | <0.001 |
| Other | 1.06 | 0.84-1.22 | 0.6 |
| Socioeconomic level |  |  |  |
| Low | Reference |  |  |
| Medium | 1.1 | 1.03-1.18 | 0.005 |
| High | 0.84 | 0.75-0.93 | 0.4 |
| Postoperative weight percentile | 1 | 1-1 | 0.8 |

**Table S13: Multivariable Poisson Regression Analysis of Lower Respiratory Tract Infection Occurrences Postoperatively**

| **Characteristic** | **RR** | **95% CI** | **p-value** |
| --- | --- | --- | --- |
| CP | 1.94 | 1.14-3.06 | 0.008 |
| Male sex | 1.06 | 1.01-1.12 | 0.029 |
| Ethnicity |  |  |  |
| Jew | Reference |  |  |
| Arab | 1.03 | 0.96-1.11 | 0.4 |
| Other | 0.99 | 0.77-1.24 | >0.9 |
| Socioeconomic level |  |  |  |
| Low | Reference |  |  |
| Medium | 0.96 | 0.89-1.03 | 0.2 |
| High | 0.66 | 0.59-0.74 | <0.001 |
| Postoperative weight percentile | 1 | 1-1 | 0.008 |

**Table S14: Multivariable Poisson Regression Analysis of Upper Respiratory Tract Infection Occurrences Postoperatively**

| **Characteristic** | **RR** | **95% CI** | **p-value** |
| --- | --- | --- | --- |
| CP | 0.77 | 0.56-1.02 | 0.087 |
| Male sex | 1.09 | 1.07-1.11 | <0.001 |
| Ethnicity |  |  |  |
| Jew | Reference |  |  |
| Arab | 1.12 | 1.09-1.15 | <0.001 |
| Other | 0.75 | 0.67-0.83 | <0.001 |
| Socioeconomic level |  |  |  |
| Low | Reference |  |  |
| Medium | 0.95 | 0.92-0.97 | <0.001 |
| High | 0.81 | 0.78-0.84 | <0.001 |
| Postoperative weight percentile | 1 | 1-1 | <0.001 |

**Table S15: Multivariable Poisson Regression Analysis of Acute Otitis Media Occurrences Postoperatively**

| **Characteristic** | **RR** | **95% CI** | **p-value** |
| --- | --- | --- | --- |
| CP | 9.47 | 5.32-15.4 | <0.001 |
| Male sex | 1.01 | 0.89-1.13 | >0.9 |
| Ethnicity |  |  |  |
| Jew | Reference |  |  |
| Arab | 0.82 | 0.69-0.98 | 0.034 |
| Other | 1.96 | 1.35-2.74 | <0.001 |
| Socioeconomic level |  |  |  |
| Low | Reference |  |  |
| Medium | 1.27 | 1.09-1.49 | 0.002 |
| High | 1.03 | 0.82-1.3 | 0.8 |
| Postoperative weight percentile | 1 | 1-1 | 0.015 |

**Table S16: Multivariable Poisson Regression Analysis of Gastroenteritis** **Occurrences Postoperatively**

| **Characteristic** | **RR** | **95% CI** | **p-value** |
| --- | --- | --- | --- |
| CP | 1.56 | 0.88-2.53 | 0.1 |
| Male sex | 1.15 | 1.09-1.21 | <0.001 |
| Ethnicity |  |  |  |
| Jew | Reference |  |  |
| Arab | 1.15 | 1.08-1.23 | <0.001 |
| Other | 1.25 | 1.02-1.51 | 0.025 |
| Socioeconomic level |  |  |  |
| Low | Reference |  |  |
| Medium | 1.19 | 1.11-1.27 | <0.001 |
| High | 0.77 | 0.68-0.86 | <0.001 |
| Postoperative weight percentile | 1 | 1-1 | >0.9 |

**Table S17: Multivariable Poisson Regression Analysis of Urinary Tract Infection** **Occurrences Postoperatively**

| **Characteristic** | **RR** | **95% CI** | **p-value** |
| --- | --- | --- | --- |
| CP | 1.16 | 0.36-2.71 | 0.8 |
| Male sex | 0.05 | 0.04-0.006 | <0.001 |
| Ethnicity |  |  |  |
| Jew | Reference |  |  |
| Arab | 1.3 | 1.17-1.44 | <0.001 |
| Other | 1.03 | 0.73-1.42 | 0.8 |
| Socioeconomic level |  |  |  |
| Low | Reference |  |  |
| Medium | 1.33 | 1.2-1.47 | <0.001 |
| High | 0.97 | 0.83-1.13 | 0.7 |
| Postoperative weight percentile | 1 | 1-1 | <0.001 |

**Table S18: Multivariable Poisson Regression Siblings Analysis of Pneumonia Occurrences Preoperatively**

| **Characteristic** | **RR** | **95% CI** | **p-value** |
| --- | --- | --- | --- |
| CP | 7.07 | 2.48-29.8 | 0.001 |
| Male sex | 0.41 | 0.2-0.8 | 0.01 |
| Preoperative weight percentile | 0.98 | 0.96-0.99 | 0.01 |

**Table S19: Multivariable Poisson Regression Siblings Analysis of Lower Respiratory Tract Infection Occurrences Preoperatively**

| **Characteristic** | **RR** | **95% CI** | **p-value** |
| --- | --- | --- | --- |
| CP | 1.3 | 0.78-2.19 | 0.3 |
| Male sex | 1.4 | 0.87-2.3 | 0.2 |
| Preoperative weight percentile | 1 | 0.99-1.01 | 0.5 |

**Table S20: Multivariable Poisson Regression Siblings Analysis of Upper Respiratory Tract Infection Occurrences Preoperatively**

| **Characteristic** | **RR** | **95% CI** | **p-value** |
| --- | --- | --- | --- |
| CP | 1.1 | 0.78-1.57 | 0.6 |
| Male sex | 0.82 | 0.59-1.13 | 0.2 |
| Preoperative weight percentile | 0.99 | 0.98-0.99 | <0.001 |

**Table S21: Multivariable Poisson Regression Siblings Analysis of Acute Otitis Media Occurrences Preoperatively**

| **Characteristic** | **RR** | **95% CI** | **p-value** |
| --- | --- | --- | --- |
| CP | 1.1 | 0.19-6.35 | >0.9 |
| Male sex | 0.44 | 0.06-2.27 | 0.3 |
| Preoperative weight percentile | 1.01 | 0.98-1.04 | 0.4 |

**Table S22: Multivariable Poisson Regression Siblings Analysis of Gastroenteritis Occurrences Preoperatively**

| **Characteristic** | **RR** | **95% CI** | **p-value** |
| --- | --- | --- | --- |
| CP | 0.63 | 0.31-1.29 | 0.2 |
| Male sex | 0.76 | 0.38-1.49 | 0.4 |
| Preoperative weight percentile | 0.99 | 0.98-1 | 0.2 |

**Table S23: Multivariable Poisson Regression Siblings Analysis of Urinary Tract Infection Occurrences Preoperatively**

| **Characteristic** | **RR** | **95% CI** | **p-value** |
| --- | --- | --- | --- |
| CP | 1.25 | 0.51-3.27 | 0.6 |
| Male sex | 0.56 | 0.23-1.31 | 0.2 |
| Preoperative weight percentile | 0.99 | 0.97-1.01 | 0.2 |

**Table S24: Multivariable Poisson Regression Siblings Analysis of Pneumonia Occurrences Postoperatively**

| **Characteristic** | **RR** | **95% CI** | **p-value** |
| --- | --- | --- | --- |
| CP | 1.44 | 0.49-4.75 | 0.5 |
| Male sex | 0.52 | 0.17-1.51 | 0.2 |
| Postoperative weight percentile | 0.98 | 0.96-1 | 0.15 |

**Table S25: Multivariable Poisson Regression Siblings Analysis of Lower Resoiratory Tract Infection Occurrences Postoperatively**

| **Characteristic** | **RR** | **95% CI** | **p-value** |
| --- | --- | --- | --- |
| CP | 1.73 | 0.56-6.45 | 0.4 |
| Male sex | 0.58 | 0.19-1.75 | 0.3 |
| Postoperative weight percentile | 0.98 | 0.95-1 | 0.083 |

**Table S26: Multivariable Poisson Regression Siblings Analysis of Upper Resoiratory Tract Infection Occurrences Postoperatively**

| **Characteristic** | **RR** | **95% CI** | **p-value** |
| --- | --- | --- | --- |
| CP | 0.73 | 0.42-1.25 | 0.2 |
| Male sex | 0.88 | 0.51-1.51 | 0.6 |
| Postoperative weight percentile | 0.99 | 0.98-1 | 0.027 |

**Table S27: Multivariable Poisson Regression Siblings Analysis of Gastroenteritis Occurrences Postoperatively**

| **Characteristic** | **RR** | **95% CI** | **p-value** |
| --- | --- | --- | --- |
| CP | 2.07 | 0.56-9.76 | 0.3 |
| Male sex | 0.49 | 0.12-1.74 | 0.3 |
| Postoperative weight percentile | 1 | 0.97-1.02 | 0.7 |

**Table S28: Excluded Records Divided into Study Groups**

| **Characteristic** | **CP** | **Control** | **p-value** |
| --- | --- | --- | --- |
| Excluded n (%) | 10 (5.7) | 4,958 (3.5) | 0.11 |
| Decease n (%) | 4 (2.3) | 270 (0.2) | <0.001 |
| Left Clalit Health Services n (%) | 6 (3.4) | 4,688 (3.3) | >0.9 |
